# Trauma-Informed Care Approach During Pediatric Venipuncture: Pre–Post Associations with Fear and Heart Rate

**DOI:** 10.3390/children13070843

**Published:** 2026-06-23

**Authors:** Emel Isıyel, Nur Mutlu, Gülay Çakmak, Özlem Tekşam

**Affiliations:** 1 Department of Pediatrics, Faculty of Medicine, Hacettepe University, Ankara 06230, Türkiye; eisiyel@gmail.com (E.I.); oteksam@yahoo.com (Ö.T.); 2 Beytepe Preschool, Hacettepe University, Ankara 06800, Türkiye

**Keywords:** trauma-informed care, venipuncture, needle phobia, procedural fear, pediatric pain, behavioral distress, heart rate, blood drawing

## Abstract

**Highlights:**

**What are the main findings?**

- A structured TIC-based intervention significantly reduced behavioral distress (escape attempts, shouting, and aggressive behaviors) during venipuncture in children with prior needle-related trauma.
- Heart rate decreased significantly after TIC (113.6 → 87.3 beats/min), and the proportion of children experiencing high levels of fear dropped from 96.2% to 15.5%.

**What is the implication of the main finding?**

- TIC-based preparation before venipuncture offers a feasible, non-pharmacological approach to managing needle-related trauma in children.
- Pediatric healthcare settings should consider incorporating trauma-informed approaches and dedicated preparation spaces into routine procedural care.

**Abstract:**

Background: Needle-related procedures such as venipuncture can be distressing for children and may trigger severe fear and behavioral dysregulation, particularly in those with previous traumatic experiences. Trauma-informed care (TIC) is a framework that recognizes the widespread impact of trauma and integrates this knowledge into clinical practice to prevent re-traumatization and support emotional regulation during medical procedures. Methods: This before-and-after study included 135 children aged 4–8 years who had previously shown severe distress during venipuncture, including escape attempts, shouting, or self/other-directed aggressive behaviors. Before venipuncture, children and their families received a TIC-based intervention delivered by a psychological counselor in a dedicated preparation room. Fear, behavioral responses during venipuncture, procedural pain, and heart rate were evaluated before and after the intervention using parent reports, the Children’s Fear Scale, the Wong–Baker FACES Pain Rating Scale, and pulse oximetry. Results: Following the TIC intervention, significant pre–post reduction were observed in distress-related behaviors during venipuncture, including escape attempts, shouting/crying, and self-/other-directed harmful behaviors. The proportion of children rated as experiencing high levels of fear decreased from 96.2% before the intervention to 15.5% after. Among the 85 children with complete heart-rate measurements available, mean heart rate decreased from 113.6 ± 10.1 beats/min to 87.3 ± 8.43 beats/min. Many families reported a more positive venipuncture experience compared with previous procedures. Conclusions: A trauma-informed care intervention delivered before venipuncture is associated with meaningful reductions in behavioral distress, fear, and physiological arousal in children with prior needle-related traumatic experiences. These pre–post associations support the feasibility and potential value of the TIC model, though controlled studies are needed to confirm these findings without confounding clinical effects.

## 1. Introduction

Illnesses and injuries are among the most common sources of emotional trauma for children and their families. Painful procedures encountered in routine medical care—such as blood collection, vascular access, and vaccination—are inherently stressful and can cause lasting emotional trauma for both children and their caregivers [1].

Emotional trauma experienced during such procedures is also a significant challenge for healthcare personnel working with children. Some children and adolescents perceive needle-related procedures as extremely alarming and painful, which can occasionally result in inability to complete the procedure. Needle-related pain and phobia may carry serious health consequences, including avoidance of pain reporting, reduced vaccine uptake, and delayed or refused treatment [2,3,4].

A large number of children are seen in hospitals and health centers every year, and repeated medical encounters increase cumulative exposure to potentially traumatic events. In recent years, many centers have shifted toward a family-centered model of care to better support children, families, and healthcare providers alike.

Trauma-informed care (TIC) is an evidence-based framework that recognizes the widespread impact of trauma and integrates this awareness into clinical practice. In this context, “trauma” refers to emotional rather than physical injury. The four core principles of TIC include: (1) recognizing the widespread impact of trauma; (2) understanding how trauma affects children, healthcare personnel, and systems; (3) responding by applying trauma-informed knowledge to practice; and (4) actively working to prevent re-traumatization. TIC approaches aim to anticipate and mitigate potential traumatic experiences arising during routine medical care, and it is well established that thoughtful management of negative procedural experiences has lasting positive effects on children’s well-being [5,6,7,8]. In pediatric care, preventing re-traumatization requires a fundamental shift in clinical perspective, where providers focus directly on identifying and reducing the distressing elements of the medical procedure itself [9]. Venipuncture can be highly distressing for children, particularly those who have had previous negative experiences with needle-related procedures. Sensory cues such as the sight of needles, the smell of antiseptics, or memories of earlier procedures may increase fear and anxiety even before the procedure begins [10]. Therefore, preparation strategies should be tailored to the child’s developmental level and emotional needs. Approaches such as therapeutic medical play, familiarization with medical equipment, relaxation exercises, distraction techniques, and caregiver support may help children feel more prepared and secure during the procedure [11,12,13]. By addressing sources of fear and promoting a sense of predictability and control, these interventions may reduce distress and support a more positive healthcare experience [9,10].

The aim of this study was to evaluate whether a structured trauma-informed care intervention delivered before venipuncture could reduce fear, behavioral distress, and physiological arousal in children with previous needle-related traumatic experiences.

## 2. Methods

### 2.1. Study Design and Participants

This before-and-after study recruited participants consecutively between 1 April 2021, and 30 October 2021, at an outpatient blood collection unit. Eligible participants were children aged 4–8 years who required venipuncture and had a history of severe distress during previous needle-related procedures. Severe distress was defined as caregiver-reported or documented escape attempts, shouting, or aggressive behaviors such as hitting, pushing, spitting, or lying on the floor.

Children with known neurodevelopmental disorders or acute medical instability requiring immediate resuscitation were excluded. All eligible children whose legal guardians provided written informed consent were consecutively enrolled during the study period.

### 2.2. Intervention

Eligible children and their families were referred to a dedicated preparation room to meet with the study’s psychological counselor. To ensure consistency across participants, all interventions were delivered by the same counselor, who held a degree in Guidance and Psychological Counseling and had certified training in trauma-informed and trauma-focused counseling approaches. The trauma-informed care (TIC) intervention followed a flexible, individualized approach rather than a fixed protocol. Session duration and intervention components were adapted according to each child’s developmental level, emotional needs, behavioral readiness, and coping capacity.

Intervention strategies included therapeutic storytelling, familiarization with medical equipment, lavender scent exposure, balloon breathing exercises, soap bubbles, drawing activities, play-based techniques, brief physical activities, cold-water face washing, drinking water, rhythmic bilateral knee tapping, and caregiver coaching. The selection of techniques was guided by the child’s preferences and responses during the session.

To increase familiarity with the procedure, the counselor demonstrated venipuncture using a doll and real medical materials, including syringes, tourniquets, and alcohol wipes. Children were encouraged to observe and handle the equipment, and individual fears and sources of discomfort were identified and addressed during the session.

Children and caregivers were also instructed in coping strategies to be used before and during venipuncture, including breathing exercises and supportive communication techniques. Venipuncture was performed only when the child indicated readiness to proceed. Following the procedure, children met briefly with the counselor to discuss their experience and reinforce successful coping strategies.

### 2.3. Outcome Measures

During the session, the psychological counselor assessed the children’s sociodemographic characteristics and explored the factors underlying their procedure-related fear or phobia. Caregivers completed study forms before and after the intervention using identical standardized instructions. Fear was assessed using the Children’s Fear Scale (CFS), and procedural pain was assessed using the Wong–Baker FACES Pain Rating Scale (WB-FACES). The CFS is a five-face scale ranging from 0 (no fear) to 4 (severe fear) and was completed by caregivers before and after the TIC intervention. Procedural pain was evaluated using the WB-FACES scale, which ranges from 0 (no pain) to 5 (worst possible pain) [14,15]. For the CFS, scores of 0–1 were categorized as little or no fear, 2 as moderate fear, and 3–4 as high fear. For the Wong-Baker FACES Pain Rating Scale, scores of 0–1 were categorized as little or no pain, 2–3 as moderate pain, and 4–5 as high pain. We have clarified these definitions in the revised manuscript.

Heart rate was measured immediately before and after the intervention using pulse oximetry by a nurse in the preparation room. Complete paired heart-rate data were available for 85 children. Missing measurements resulted from technical difficulties during the procedure, primarily motion artifacts and probe displacement.

To assess the broader effects of the intervention, behavioral indicators of distress and avoidance were documented before and after the intervention. Caregivers reported whether the child attempted to escape, engaged in self-harming behaviors, cried before entering the procedure room, or cried during needle insertion. Additional indicators of procedural distress, including the need for physical restraint, being forced into the blood collection chair, or exhibiting marked physical resistance, were also recorded.

In addition, caregivers completed a study-specific questionnaire consisting of predefined, closed-ended items designed to evaluate their perceptions of the intervention and its perceived effects on the child’s ability to cope with venipuncture.

Before hospital discharge, children met again with the psychological counselor to discuss their experience and perceived benefits of the intervention. Overall, the TIC intervention was designed to prepare children and their families for venipuncture in a psychologically supportive environment and to reduce the risk of re-traumatization.

### 2.4. Statistical Analysis

A formal a priori sample size calculation was not performed; instead, we enrolled all eligible participants available during the study period. Statistical analyses were performed using IBM SPSS Statistics for Windows, version 26.0 (IBM Corp., Armonk, NY, USA). Continuous and ordinal variables were initially evaluated for distributional properties using the Shapiro–Wilk test, supplemented by visual inspection of histograms and Q-Q plots. Because the pre- and post-intervention clinical scores and physiological data demonstrated non-normal distributions, non-parametric statistical methods were systematically preferred. Pre–post comparisons for continuous and ordinal outcomes were analyzed using the Wilcoxon signed-rank test. For paired categorical clinical variables, the McNemar–Bowker test was utilized. All reported *p*-values were two-sided, and a *p*-value of less than 0.05 was considered statistically significant [16].

## 3. Results

A total of 135 children (81 boys, 54 girls) were included in the study. The mean age of the participants was 5.4 ± 1.5 years, with a minimum age of 4 and a maximum age of 8 years. The most commonly reported sources of fear were needle insertion (131 children, 97.0%), tourniquet application (128 children, 94.8%), sight of blood (24 children, 17.7%), and the sensation of a foreign object (14 children, 10.3%).

Behavioral responses during venipuncture improved significantly following the TIC intervention across all assessed domains. The proportion of children rated as “too much” for escape attempts decreased from 64.4% to 2.9%, shouting or crying from 88.1% to 8.1%, and self-/other-directed aggressive behaviors from 22.2% to 0.7%. Detailed pre- and post-intervention comparisons for all behavioral outcomes are presented in Table 1, with statistically significant differences observed for all items (*p* < 0.01).

Heart rate measurements were available for 85 children. Mean heart rate decreased significantly from 113.6 ± 10.1 beats/min (median: 119 beats/min) before the intervention to 87.3 ± 8.43 beats/min (median: 92 beats/min) after the intervention (*p* < 0.01). Fear levels also improved markedly: the proportion of children rated as having high levels of fear decreased from 96.2% before the intervention to 15.5% after, while the proportion rated as having moderate levels of fear increased from 2.2% to 62.9% (*p* < 0.01). These findings are summarized in Table 2.

**Figure 1:**
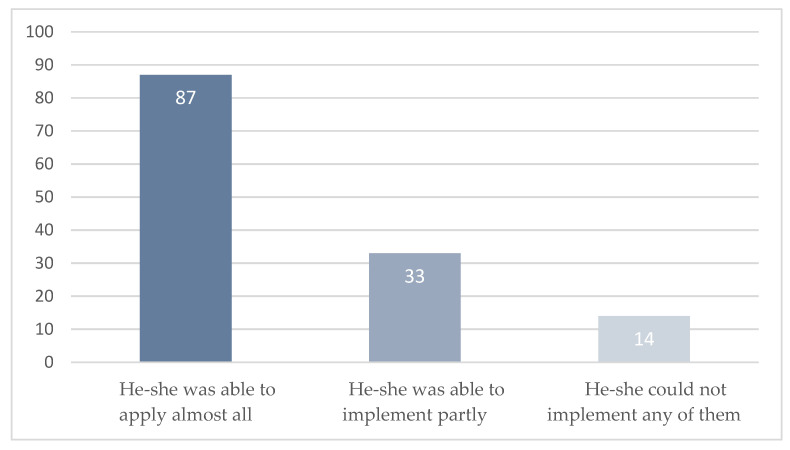
Children’s ability to apply TIC.

**Figure 2:**
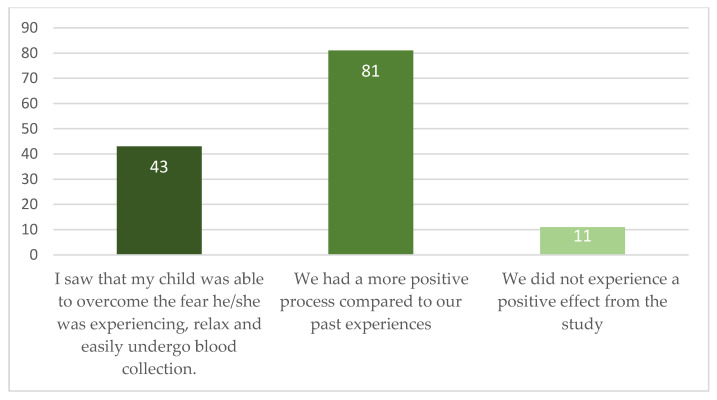
Evaluation of TIC implementation by families.

Regarding procedural pain, 42.2% of children experienced little or no pain during venipuncture after the TIC intervention, while 25.1% reported moderate pain and 32.5% reported significant pain (Table 3). The majority of children (64.4%) were able to apply almost all of the TIC techniques during the procedure, while 24.4% applied them partially and 10.3% were unable to apply any coping strategies (Figure 1). Overall, 91.8% of families reported a positive experience compared with previous venipuncture encounters (Figure 2). Venipuncture was completed on the first attempt in 131 out of 135 children (97.0%), while only four children (3.0%) required a second attempt.

## 4. Discussion

Negative experiences during childhood, including those occurring in healthcare settings, may have lasting effects on both physical and mental health outcomes [8]. Early recognition and mitigation of these experiences remain important public health priorities, and trauma-informed care (TIC) has emerged as a structured framework to support this goal within pediatric healthcare systems [7,8]. In the present study, children with previous needle-related traumatic experiences showed significant reductions in behavioral distress, anxiety, and heart rate following a structured TIC-based intervention delivered before venipuncture.

Previous research has shown that psychological interventions can be beneficial in reducing needle-related pain and distress in children and adolescents. In a Cochrane systematic review, Birnie et al. reported beneficial effects across a range of psychological approaches, with distraction-based techniques being the most frequently studied interventions [15]. However, evidence specifically addressing children with pre-existing needle-related trauma remains limited. Unlike conventional distraction strategies, our intervention combined exposure-based familiarization, somatic regulation techniques, and caregiver coaching. This approach was designed not only to support children during the procedure itself but also to address the impact of previous traumatic experiences. Consistent with this perspective, McMurtry et al. emphasized that exposure-based interventions may be particularly beneficial for children with pronounced needle fear or phobia [4].

The reduction in heart rate observed after the intervention suggests a decrease in physiological arousal during venipuncture. Similar observations have been reported in studies using virtual reality-based preparation and mindfulness interventions, both of which were associated with better emotional regulation and lower physiological stress responses during pediatric procedures [16,17,18,19].

Although heart rate is influenced by multiple factors, the parallel reductions in behavioral and emotional distress observed in our study support the interpretation that children experienced lower levels of procedural stress following the intervention. The substantial reduction in fear levels, with the proportion of children classified as having high fear decreasing from 96.2% to 15.5%, further highlights the potential clinical relevance of the TIC approach. These findings are consistent with previous reports indicating that psychological interventions can improve children’s procedural experiences and reduce distress during painful medical procedures [1]. Likewise, the importance of providing a safe, child-centered environment and ensuring that healthcare professionals are adequately trained to support children during invasive procedures has been emphasized in earlier pediatric pain literature [2]. In our study, the intervention was delivered in a sensory-friendly setting that included environmental modifications such as lavender aroma diffusion. Previous research has suggested that lavender-based aromatherapy may reduce procedural anxiety and pain in children undergoing medical or dental procedures [13]. However, our study was not designed to evaluate the independent effects of aromatherapy, and therefore its specific contribution to the observed outcomes cannot be determined.

From a practical perspective, this TIC-based model requires minimal material resources, relying primarily on inexpensive items such as drawing materials, soap bubbles, and standard clinical equipment. Although individualized preparation requires additional staff time and may be challenging in high-volume outpatient settings, efforts to reduce procedural distress remain important. Positive procedural experiences during childhood may contribute to better cooperation in future healthcare encounters and may reduce the risk of developing persistent healthcare-related fears. Institutional strategies such as dedicated preparation areas and staff training may facilitate implementation of trauma-informed practices in pediatric settings. Future studies should also evaluate whether similar benefits can be achieved in children undergoing their first venipuncture experience.

Several limitations should be considered when interpreting the findings of this study. First, the absence of a control group in this before-and-after study limits causal inference. Although substantial improvements were observed following the intervention, these changes cannot be attributed solely to the trauma-informed care model. Factors such as natural habituation, parental expectations, and the additional attention provided by the psychological counselor may also have contributed to the observed improvements. Furthermore, because many participants entered the study with high levels of fear and distress, some improvement may reflect regression toward the mean rather than a true intervention effect. Parent-reported outcomes may also have been influenced by expectancy bias, whereby parents who anticipated benefit from the intervention perceived their children’s experiences more positively. Second, complete pre- and post-intervention heart rate measurements were available for only 85 of the 135 participants. Because this missing data was largely due to motion artifacts and probe displacement from children’s physical movements, the missingness may be linked to higher baseline distress, potentially introducing selection bias. Third, behavioral, fear, and pain outcomes were assessed exclusively through parent proxy-reports. Although child self-report is generally considered the preferred method for evaluating subjective experiences, obtaining reliable self-assessments from children aged 4–8 years immediately after a stressful venipuncture procedure can be difficult. Given the age range of our study population, parent proxy reports represented a practical and clinically meaningful alternative, although they remain susceptible to reporting bias. Fourth, the intervention was delivered by a single trained psychological counselor at a single center, which may limit the generalizability of the findings. Finally, no long-term follow-up assessments were performed, and it remains unclear whether the observed benefits persist during subsequent venipuncture procedures. In addition, multiple behavioral, pain, and physiological outcomes were evaluated. Therefore, the findings should be interpreted as exploratory and hypothesis-generating rather than definitive causal conclusions.

## 5. Conclusions

This study demonstrates that a structured trauma-informed care (TIC) intervention delivered before venipuncture was associated with significant reductions in behavioral distress, fear, and heart rate among children with prior needle-related traumatic experiences. Consistent with these positive pre–post changes, 131 of 135 children successfully employed the coping strategies and completed the procedure on the first attempt, while families consistently reported a more positive experience compared with previous healthcare encounters. These findings suggest that TIC-based preparation, delivered by a trained psychological counselor in a dedicated setting, represents a feasible and promising non-pharmacological approach for managing procedural distress in pediatric care.

Given the large number of children who undergo invasive procedures each year and the potential long-term consequences of unaddressed medical trauma, the integration of trauma-informed approaches into routine pediatric practice deserves careful consideration. Future studies using randomized controlled designs are needed to confirm these findings, identify the specific contributions of individual intervention components, and determine whether the observed benefits are sustained across subsequent healthcare encounters.

## Figures and Tables

**Table 1:** Comparison of children’s behavioral responses before and after the TIC intervention.

	Pre-TIC			Post-TIC			Somers’ d [95% CI]	*p* Values
	Too Much (*n*/%)	Little Bit (*n*/%)	No (*n*/%)	Too Much (*n*/%)	Little Bit (*n*/%)	No (*n*/%)		
Trying to escape to avoid blood collection	87 (64.4%)	18 (13.3%)	30 (22.2%)	4 (2.9%)	9 (6.6%)	122 (90.3%)	0.720|[0.638, 0.802]	<0.01
Shouting, crying to avoid blood collection	119 (88.1%)	11 (8.1%)	5 (3.7%)	11 (8.1%)	18 (13.3%)	106 (78.5%)	0.859|[0.796, 0.922]	<0.01
Harming yourself and others to prevent blood collection (hitting, pushing, lying down)	30 (22.2%)	15 (11.1%)	90 (66.6%)	1 (0.7%)	8 (5.9%)	126 (93.3%)	0.279|[0.189, 0.369]	<0.01
Being forced to sit on the blood collection seat	126 (93.3%)	7 (5.1%)	2 (1.4%)	38 (28.1%)	69 (51.1%)	28 (20.7%)	0.655|[0.569, 0.741]	<0.01
Grabbing his/her arm on the blood collection seat	111 (82.2%)	20 (14.8%)	4 (2.9%)	42 (31.2%)	12 (8.8%)	81 (60%)	0.597|[0.501, 0.693]	<0.01
Overstraining during blood collection	124 (91.8%)	10 (7.5%)	1 (0.7%)	25 (18.5%)	78 (57.7%)	32 (23.8%)	0.747|[0.669–0.825]	<0.01
Starting to cry before entering the blood collection room	88 (65.1%)	28 (20.7%)	19 (14%)	66 (48.8%)	14 (10.3%)	8 (5.9%)	−0.102|[−0.224–0.02]	<0.01
Starting to cry in the blood collection room	114 (84.4%)	13 (9.6%)	6 (4.4%)	10 (7.5%)	23 (17%)	81 (60%)	0.716|[0.669–0.825]	<0.01
Starting to cry when seeing the needle	131 (97%)	2 (1.4%)	2 (1.4%)	74 (54.8%)	35 (25.9%)	24 (17.7%)	0.62|[0.511–0.734]	<0.01
Crying throughout the process	111 (82.2%)	20 (14.9%)	4 (2.9%)	18 (13.3%)	32 (23.7%)	85 (62.9%)	0.68|[0.581–0.782]	<0.01
Crying when the needle enters, but then calming down	4 (2.9%)	27 (20%)	104 (77%)	88 (65.1%)	28 (20.7%)	19 (14%)	0.59|[0.48–0.701]	<0.01

CI, Confidence Interval. Somers’ d was calculated as an asymmetric measure of effect size.

**Table 2:** The evaluation of fear levels and pulse rates before and after TIC in children who experienced trauma before or during blood collection.

	Pre-TIC	Post-TIC	*p* Value
Fear level (*n*:135)			
Little	2 (1.4%)	28 (20.7%)	<0.01
Medium	3 (2.2%)	85 (62.9%)	<0.01
Much	130 (96.2%)	21 (15.5%)	<0.01
Pulse rate (*n*:85) (mean and SD)	113.6 ± 10.1	87.3 ± 8.43	<0.01

**Table 3:** The pain levels felt by the patients during the procedure.

Pain During the Procedure	*n* (%)
Little or no	57 (42.2)
Medium	34 (25.1)
Much	44 (32.5)

## Data Availability

The data presented in this study are available upon reasonable request from the corresponding author.
